# Fabrication and Measurement of a Suspended Nanochannel Microbridge Resonator Monolithically Integrated with CMOS Readout Circuitry

**DOI:** 10.3390/mi7030040

**Published:** 2016-03-02

**Authors:** Gabriel Vidal-Álvarez, Eloi Marigó, Francesc Torres, Núria Barniol

**Affiliations:** Department of Electronic Engineering, Universitat Autònoma de Barcelona (UAB), Cerdanyola del Vallès 08193, Spain; gabriel.vidal@uab.es (G.V.-Á.); eloi.marigo@uab.es (E.M.); francesc.torres@uab.es (F.T.)

**Keywords:** nanochannel, suspended channel, resonators, CMOS-NEMS, NEMS

## Abstract

We present the fabrication and characterization of a suspended microbridge resonator with an embedded nanochannel. The suspended microbridge resonator is electrostatically actuated, capacitively sensed, and monolithically integrated with complementary metal-oxide-semiconductor (CMOS) readout circuitry. The device is fabricated using the back end of line (BEOL) layers of the AMS 0.35 μm commercial CMOS technology, interconnecting two metal layers with a contact layer. The fabricated device has a 6 fL capacity and has one of the smallest embedded channels so far. It is able to attain a mass sensitivity of 25 ag/Hz using a fully integrable electrical transduction.

## 1. Introduction

Micro and nanoelectromechanical systems (MEMS and NEMS) have been largely studied to sense different kind of magnitudes, such as mass, pressure, temperature, *etc.* They have proved to be very competitive compared to other sensing technologies. Specifically, NEMS mass sensors, based on measuring the resonance frequency shift produced by the presence of a mass in some point of the resonator, have achieved mass resolutions in the range of yoctograms, achieving the single atom limit [1]. However, some mass sensing measurements require the sensors to operate in conditions where resonant MEMS or NEMS devices do not perform at their best, significantly worsening the mass resolution. For instance, some measurements of biological samples need to be carried out in an aqueous environment, reducing the quality factor of the device and, thus, its mass resolution [2].

To overcome this problem, Burg and Manalis presented a device known as the suspended microchannel resonator (SMR), consisting of a cantilever with an embedded microfluidic channel [3]. This configuration allows the device to operate in a vacuum environment, since the analytes—the mass of which we want to measure—flow through the resonator using the embedded microfluidic channel. Due to the fact that the device is operated in vacuum, the quality factor does not decrease significantly and a high mass resolution can be achieved.

The Burg and Manalis SMR shown in Reference [3] consisted of a cantilever with one or several embedded microfluidic channels. The channels dimensions were up to 500 μm long and had a height of 500 nm. For the specific case of the 300 μm long cantilever, the SMR had a capacity of 27 pL and a mass sensitivity lower than 10 pg/Hz. The cantilever was actuated electrostatically and its deflection was measured employing the optical lever method. Since then, a huge variety of SMRs, aiming to improve the mass sensitivity, have been proposed, presenting a variety of channel dimensions, geometries, and transduction principles. For instance, following Burg’s approach, Reference [4] showed a cantilever with a capacity around 140 fL and a mass sensitivity of 840 ag/Hz. Similarly, in Reference [5], an even better device, with a capacity of 22 fL and a mass sensitivity of 107 ag/Hz, was presented. In this last case, the actuation was piezoelectric instead of electrostatic. In Reference [6], completely different geometry and transduction principles were employed. Specifically, suspended bridge resonators, containing channels of nanoscale dimensions with optothermal actuation and Fabry-Perot interferometry optical detection, were demonstrated, achieving a mass sensitivity of around 10 ag/Hz in a 4 fL capacity channel. In Reference [7], piezoresistive detection was employed, enabling the possibility of integrated SMRs. However, in this case, the SMR was much bigger—it was a microscale cantilever containing a bidirectional channel with a capacity of around 26.5 pL—and had a mass sensitivity of 180 fg/Hz. Bulk-mode resonators with embedded microchannels and integrable transduction—electrostatic actuation and capacitive detection—were employed in Reference [8], achieving mass sensitivities of 855 ag/Hz in a 223 fL capacity channel. Recently, following this bulk-mode resonator approach, a 150 μm wide square-plate resonator with embedded microchannels has been reported. The resonator was similar to the one in Reference [8], but had a larger size in order to improve the transduction, and had a mass sensitivity of around 30 fg/Hz [9]. Finally, piezoelectric actuation and detection have also been demonstrated, but without providing integrable solutions [10].

This variety of devices has improved the capabilities and resolution of SMRs, demonstrating that smaller embedded nanochannels achieve better mass sensitivities. For example, in terms of mass resolution, several order-of-magnitude improvements have been achieved, reaching the attogram resolution in References [4,5,6]. However, in these cases, optical detection techniques were used, making more difficult the widespread use of these sensors due to its difficult integrability. To overcome this problem, we present a high mass sensitivity electrostatically actuated and capacitively sensed suspended microbridge resonator with an embedded nanochannel. The device is integrated in CMOS technology, providing an integrable alternative to the state-of-the-art SMRs. The fabrication of nanochannels in standard CMOS technology allows for the integration of the signal processing circuitry into the chip, easing the path to high mass resolution bio-sensing system-on-a-chip devices. The design of the suspended microbridge resonator and its embedded nanochannel is described in Section 2. Its fabrication is detailed in Section 3. The main characterization results, which validate the approach proposed, together with a comparison with the state-of-the-art of SMRs are shown in Section 4. Finally, Section 5 gives the conclusions.

## 2. Suspended Nanochannel Resonator (SNR) Design

**Figure 1:**
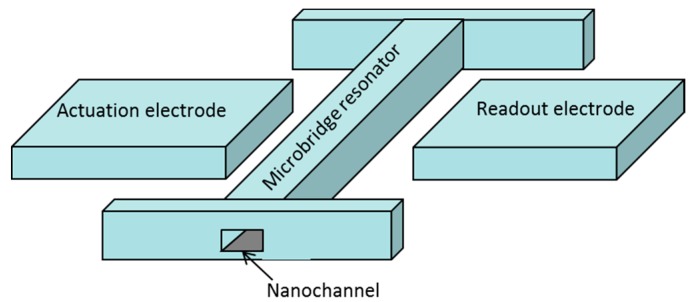
Conceptual sketch of a suspended nanochannel resonator (SNR) formed by an in-plane microbridge resonator and a nanochannel embedded inside.

The MEMS resonator designed in this work consists of a microbridge or clamped-clamped beam with a built-in nanochannel, which we will call a suspended nanochannel resonator (SNR). A sketch of the device is shown in Figure 1. The SNR is electrostatically actuated and capacitively sensed through the two electrodes placed on opposite sides of the bridge. This electrode configuration promotes a lateral movement of the bridge that corresponds to the SNR’s first flexural in-plane mode. The SNR is driven to resonance by applying an alternating current (AC) signal to one of the electrodes (actuation), while the other electrode (readout) is used for detecting the SNR’s lateral movement. The motional current, as a result of the variation of the capacitance between the bridge and the readout electrode, due to the oscillating movement, is detected and measured using a specific CMOS readout circuit, as previously reported [11]. The microbridge resonator dimensions (length and width) are the adequate to exhibit resonance frequency in the range of tens of MHz, guaranteeing full functionality of the integrated CMOS circuitry, while its thickness is defined by the technological constraints of the CMOS technology used (see Section 3).

## 3. Device Fabrication

**Figure 2:**
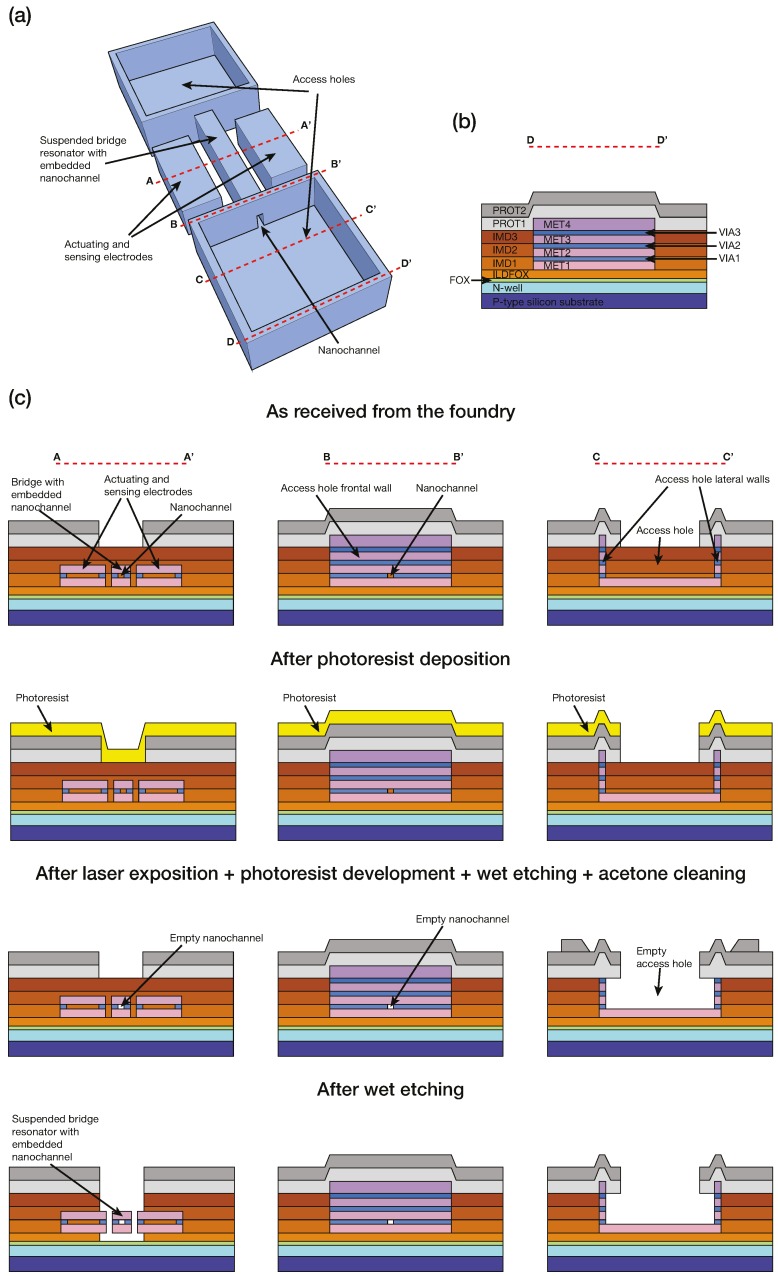
Illustrations of the suspended nanochannel microbridge resonator at different stages of the process to empty the nanochannel and release the bridge. For the sake of clarity, the size proportions of the different elements of the suspended nanochannel microbridge resonator (access holes, electrodes-resonator gaps, bridge nanochannel aperture, *etc.*) are modified compared to the actual device. (**a**) Simplified 3D illustration of the suspended nanochannel bridge resonator once empty and released. The dashed lines indicate the four different cross-sections. (**b**) Cross-section D-D’ of the fabricated suspended nanochannel bridge resonator showing all the layers of the AMS 0.35 μm commercial CMOS technology. PROT1 and PROT2 correspond to passivation layers; MET1, MET2, MET3, and MET4 to metal layers; VIA1, VIA2, and VIA3 to contact layers; FOX, ILDFOX, IMD1, IMD2, and IMD3 correspond to insulator layers. The evolution of the cross-section D-D’ is not shown, since it remains unchanged after the completion of all the carried out processes. (**c**) Cross-sections A-A’, B-B’, and C-C’ showing the bridge with the built-in nanochannel and the access holes at different stages of the emptying and releasing fabrication process.

**Figure 3:**
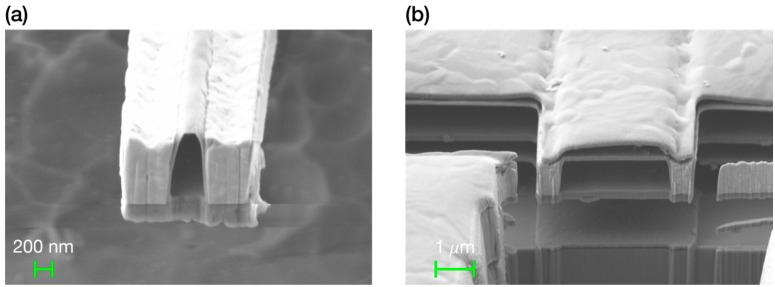
Scanning electron microscope (SEM) images after focused ion beam (FIB) milling showing suspended nanochannel microbridge resonators fabricated using different layers and presenting nanochannels of different widths. (**a**) MET3-VIA3-MET4 nanochannel of 450 nm width, the minimum width allowed by the technology. (**b**) MET2-VIA2-MET3 nanochannel of 3 μm width.

To fabricate the proposed SNR, we made use of the AMS 0.35 μm commercial CMOS technology with the specific layer structure shown in Figure 2b. In order to define the SNR device, we used a sandwiched stack, formed by two metal layers, used as bottom and top walls of the nanochannel. These two metal layers were linked together through a continuous contact layer, which confined the interlevel oxide between the metal layers and formed the sidewalls of the nanochannel. Due to the fact that the main purpose of AMS technology is to obtain CMOS integrated circuits—and not obtaining MEMS devices or nanochannels—one of the main challenges of our design was to demonstrate that the nanochannel was watertight. As we can see further in this work (see Figure 3), we have demonstrated that by using the via layer as a continuous contact layer (as opposite to the normal procedure in a CMOS circuit, where the via layer is discontinuous), nanochannel sealing is achieved.

Once the chip was received from the CMOS foundry with the defined SNR device, a dedicated etching process to erase the field and interlevel silicon dioxides was performed. Specifically, the resonator was released, etching the silicon dioxide that surrounds the stack resonator formed by the two metal layers, while the nanochannel was formed once the interlevel oxide was fully etched. At both ends of the bridge, two access holes connecting the nanochannel to the top of the chip were also defined. To fabricate the access holes, we used all metal and contact layers that rest above the ones used for the bridge resonator. We did so in order to make the system, from access hole to access hole, completely watertight.

To empty the nanochannel of silicon dioxide, we protected the chip with a positive photoresist (Microposit S1813 D1 Photo Resist from Shipley Company Inc.), defining with direct laser lithography only two apertures just above the access holes. Then, we performed wet etching with a buffered hydrogen fluoride (BHF) solution. After the BHF wet etching, we removed the photoresist with acetone. We repeated this sequence several times until the nanochannel was empty. Note that depending on the layers used to fabricate the device, we had to adjust the duration and number of repetitions of the sequence: photoresist deposition, laser exposition, photoresist development, wet etching, and acetone cleaning. Finally, without any photoresist protection, we performed a final BHF wet etching to release the bridge.

For the specific case of the device presented in this work, the MET1 and MET2 metal layers, together with the VIA1 contact layer, were used to define the microbridge with the embedded nanochannel. However, other choices of metal and contact layers, for instance MET2-VIA2-MET3 or MET3-VIA3-MET4, are also possible in order to fabricate similar devices. For the access holes, all metal (MET1, MET2, MET3, and MET4) and contact (VIA1, VIA2, and VIA3) layers were used. For the fabricated device, a MET1-MET2 stack bridge, 15 μm long and 1.45 μm wide, with a built-in nanochannel of 450 nm. With these dimensions and layers, and according to a finite element method (FEM) simulation, the expected resonance frequency for the empty SNR is 33 MHz. To empty the nanochannel, we repeated the wet etching sequence three times, with each of the wet etchings lasting 18 min. In addition, we performed two more wet etchings, without any photoresist protection, to release the structure, each lasting, respectively, 18 and 10 min.

Figure 2 shows a detailed description of the fabrication process we followed. Specifically, Figure 2a shows a 3D illustration of the suspended microbridge resonator, while Figure 2b shows the different layers used of the AMS 0.35 μm technology. Figure 2c shows different cross-sections at different steps of the process envisioned to empty the nanochannel and release the bridge.

As mentioned, different suspended nanochannel bridge resonators can be defined using the AMS 0.35 μm CMOS technology. The channel width has a minimum value of 450 nm, but other greater channel widths can be designed. The bridge thickness and the channel height are defined by the thickness of the different layers of the technology used to define the bridge. The typical thickness values of the different layers can be seen in Table 1. Figure 3 shows two examples of different suspended nanochannel bridge resonators, having different widths and fabricated using different constitutive layers.

## 4. Experimental Analysis

**Figure 4:**
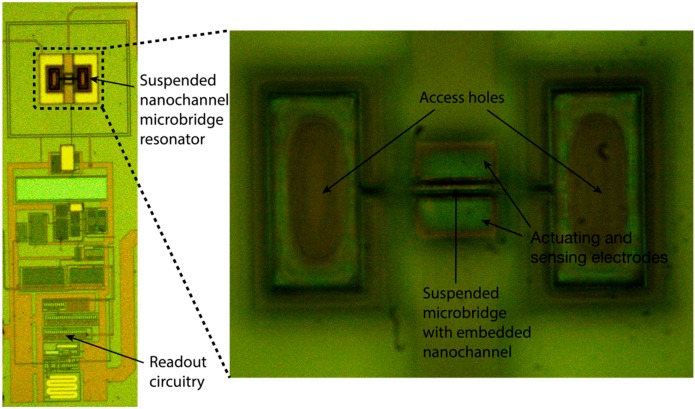
Optical image of the suspended nanochannel microbridge resonator and its adjacent CMOS readout circuitry at some intermediate step in the emptying and releasing process.

A suspended nanochannel microbridge resonator monolithically integrated in the AMS 0.35 μm CMOS technology was used to demonstrate the initial steps towards its use for mass sensing applications. The fabricated device can be seen in Figure 4, where we show an optical image of the fabricated suspended nanochannel microbridge resonator together with its CMOS readout circuitry. The CMOS readout circuitry employed was previously reported in reference [11].

**Figure 5:**
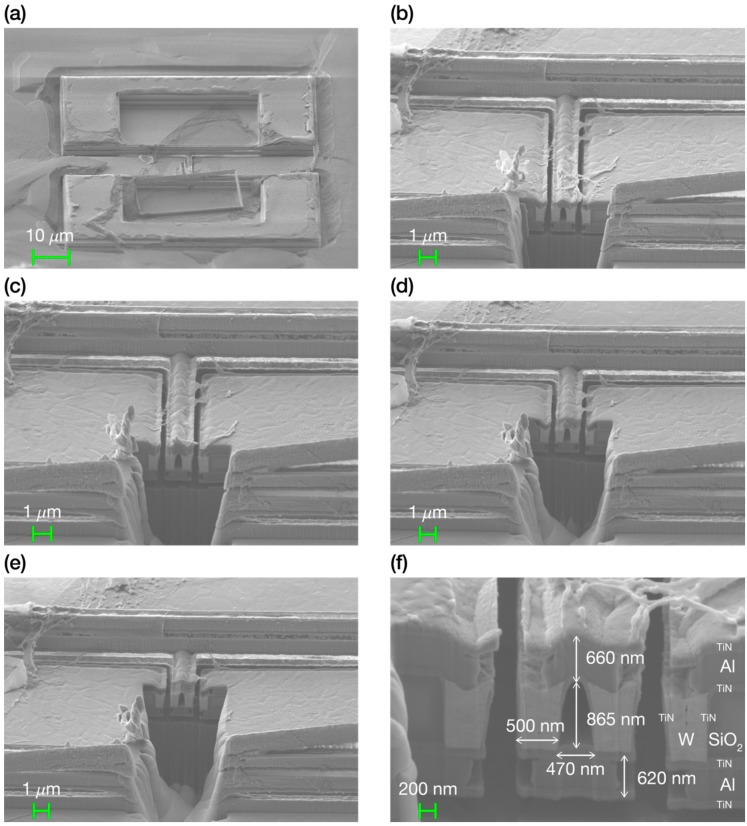
SEM images of a fabricated suspended microbridge resonator 10 μm long and 1.45 μm wide, with a built-in nanochannel of 450 nm, before and after some FIB cuts. (**a**) General view of the suspended nanochannel bridge resonator before making any FIB cut. (**b**) SEM image showing the interior of the nanochannel after a first FIB cut. We can easily appreciate that the nanochannel is empty for a quarter of its length. (**c**) SEM image showing the interior of the nanochannel after a second FIB cut. The nanochannel is empty for almost half its length. (**d**) SEM image showing the interior of the nanochannel after a third FIB cut. We see that the nanochannel is empty for more than half its length. Thus, it will be completely empty since the BHF bath etches silicon dioxide from both extremes. (**e**) SEM image showing the interior of the nanochannel after a fourth FIB cut. From the sequence of FIB cuts, we see that the nanochannel is completely empty from access hole to access hole. (**f**) SEM image showing a zoom in of the nanochannel. The cross-section image shows that the nanochannel has a width in the base of 470 nm and a height of 865 nm.

To show that the nanochannel inside the suspended bridge resonator was actually empty and the bridge released, we performed some focused ion beam (FIB) milling cuts perpendicular to the bridge main axis orientation. After each cut, we took a scanning electron microscope (SEM) image showing the interior of the nanochannel. Figure 5 shows a general view and some FIB milling cuts of the suspended nanochannel microbridge resonator, demonstrating that the nanochannel was completely empty along all its length, from access hole to access hole. As a side remark, Figure 5f show that the metal and contact layers specified by the AMS 0.35 μm technology were not homogeneous, but formed by different layers of different materials. The metal layers were sandwiches of aluminum (Al) between two layers of titanium nitride (TiN), while the contact layers were of tungsten (W) with the lateral and bottom walls confining it made of titanium nitride. Note also that the silicon dioxide (SiO_2_) confined in the electrodes, as expected, was not etched.

**Figure 6:**
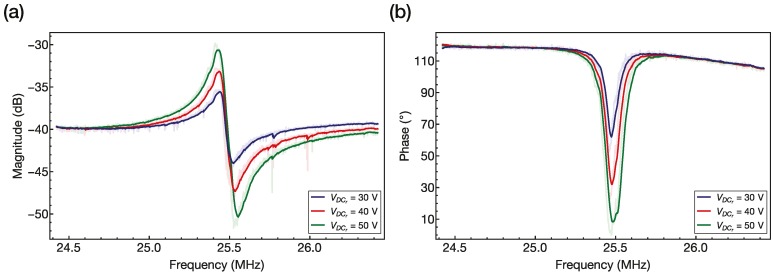
Experimental frequency response in air conditions of the capacitively detected suspended nanochannel microbridge resonator with monolithically integrated CMOS readout circuitry for different resonator DC voltages. The device has undergone the following wet etchings: 18 min + 18 min + 18 min + 10 min. (**a**) Magnitude frequency response. (**b**) Phase frequency response.

Before making the FIB milling cuts to demonstrate that the built-in nanochannel was empty, we measured the electrical frequency response of the device. The electrical characterization of the bridge-shaped SNR resonator was performed using a network analyzer (E5100A from Agilent, Santa Clara, CA, USA). The motional current generated by the resonant SNR was measured using a three-electrode configuration. A direct current (DC) voltage was applied to the bridge, an AC excitation voltage was applied to the actuation electrode, and the electrical readout signal was acquired from the readout electrode through the CMOS amplifier. Figure 6 shows three electrical measurements of the device corresponding to three different resonator DC voltages.

In Figure 6, we see that the device resonates properly—the motional current increases with the resonator DC voltage, and a small shift to lower frequencies due to electrostatic spring softening can be appreciated [12]. The measured resonance frequency of 25.4 MHz is in a similar range to that of the computed one, and the extracted quality factor is 250 in air conditions. Note that the FEM simulations were performed with a perfect rectangular shape for all the layers, which is not the actual shape according to the images shown in Figure 5. Additionally, we can perfectly see that the monolithically integrated CMOS readout circuitry amplifies the response of the resonator [11].

As can be seen in Figure 6, the fabricated device has a resonance frequency of about 25.4 MHz. Thus, estimating the mass sensitivity, as in Reference [5], *i.e.*, calculating the mass of the suspended bridge resonator from its dimensions and materials, and considering the nanochannel filled with water, our device can attain a mass sensitivity of around 25 ag/Hz, which is comparable to the mass sensitivity of around 10 ag/Hz of the device presented in Reference [6] (the most sensitive SMR yet).

Table 2 shows some of the characteristics of state-of-the-art suspended microchannel resonators, and how the device we present in this work compares to them. The devices shown in Reference [6] present the best mass sensitivities; however, the transduction mechanisms used to actuate and sense the device were not integrable. In our approach, based on the fabrication of nanochannels in standard CMOS technology, we can attain similar mass sensitivities and, at the same time, achieve integration of the signal processing circuitry into the chip, easing the path to a system-on-a-chip device with high mass resolution.

## 5. Conclusions

In this work, we presented the fabrication and electrical measurement of a microbridge resonator with a built-in nanochannel, monolithically integrated with CMOS readout circuitry. We demonstrated that it is possible to obtain a nanochannel that is completely watertight using standard CMOS technology, with no more steps than those needed to release the structure and to empty the channel. The actuation was electrostatic and the detection was capacitive, both are fully on-chip transduction methods with no additional excitation or readout systems. The combination of nanosize channel dimensions and integrated circuitry opens the possibility to attain unprecedented mass resolution, with resonant micromechanical sensors, measuring the mass of analytes, which require an aqueous environment.

## Figures and Tables

**Table 1:** Thickness of the different metal and interlevel oxide layers of the AMS 0.35 μm CMOS technology.

Layer	Typical Thickness (nm)
MET1	665
IMD1	1000
MET2	640
IMD2	1000
MET3	640
IMD3	1000
MET4	925

**Table 2:** Geometry, dimensions, channel capacity, estimated mass, resonant frequency, mass sensitivity, and types of transduction for different state-of-the-art SMRs and this work’s device. The mass and mass sensitivity were calculated considering the resonators filled with water.

Type of SMR and Year	Bridge, 2010 [6]	Cantilever, 2010 [4]	Cantilever, 2014 [5]	Bridge, 2015 (This Work)
Length (μm)	20	50	27	15
Thickness (μm)	4.3	1.3	1	2.145
Width (μm)	2.187	10	7.5	1.47
Channel height (nm)	107	700	400	865
Channel width (nm)	1850	2000	1000	470
Channel capacity (fL)	4	140	22	5.8
Mass (pg)	100	1328	443	330
Resonant frequency (MHz)	24	0.76	1.99	25.2
Mass sensitivity (ag/Hz)	8.3	840	107	25 *
Transduction	Optothermal actuation and optical detection	Electrostatic actuation and optical-lever detection	Piezoelectric actuation and optical-lever detection	Electrostatic actuation and capacitive detection

Note: * Estimated mass sensitivity.

## References

[1] Chaste J., Eichler A., Moser J., Ceballos G., Rurali R., Bachtold A. (2012). A nanomechanical mass sensor with yoctogram resolution. Nat. Nanotech..

[2] Burg T.P., Godin M., Knudsen S.M., Shen W., Carlson G., Foster J.S., Babcock K., Manalis S.R. (2007). Weighing of biomolecules, single cells and single nanoparticles in fluid. Nature.

[3] Burg T.P., Manalis S.R. (2003). Suspended microchannel resonators for biomolecular detection. Appl. Phys. Lett..

[4] Lee J., Shen W., Payer K., Burg T.P., Manalis S.R. (2010). Toward attogram mass measurements in solution with suspended nanochannel resonators. Nano Lett..

[5] Olcum S., Cermak N., Wasserman S.C., Payer K., Shen W., Lee J., Manalis S.R. Suspended nanochannel resonators at attogram precision. Proceedings of the 27th IEEE International Conference on Micro Electro Mechanical Systems (MEMS 2014).

[6] Barton R.A., Ilic B., Verbridge S.S., Cipriany B.R., Parpia J.M., Craighead H.G. (2010). Fabrication of a nanomechanical mass sensor containing a nanofluidic channel. Nano Lett..

[7] Lee J., Chunara R., Shen W., Payer K., Babcock K., Burg T.P., Manalis S.R. (2011). Suspended microchannel resonators with piezoresistive sensors. Lab Chip.

[8] Agache V., Blanco-Gomez G., Baleras F., Caillat P. (2011). An embedded microchannel in a MEMS plate resonator for ultrasensitive mass sensing in liquid. Lab Chip.

[9] Hadji C., Berthet C., Baleras F., Cochet M., Icard B., Agache V. Hollow MEMS mass sensors for real-time particles weighing and sizing from a few 10 nm to the μm scale. Proceedings of the 28th IEEE International Conference on Micro Electro Mechanical Systems (MEMS 2015).

[10] Zuniga C., Rinaldi M., Piazza G. High frequency Piezoelectric Resonant Nanochannel for bio-sensing applications in liquid environment. Proceedings of the 9th Annual IEEE Conference on Sensors (Sensors 2010).

[11] Verd J., Uranga A., Abadal G., Teva J.L., Torres F., López J., Pérez-Murano F., Esteve J., Barniol N. (2008). Monolithic CMOS MEMS oscillator circuit for sensing in the attogram range. IEEE Electron Device Lett..

[12] Teva J., Abadal G., Davis Z.J., Verd J., Borrisé X., Boisen A., Pérez-Murano F., Barniol N. (2004). On the electromechanical modelling of a resonating nano-cantilever-based transducer. Ultramicroscopy.

